# The effects of learning with various noise on Gait Kinematics in 3-to-5-year-old children: a randomized controlled trial

**DOI:** 10.1186/s13102-022-00416-2

**Published:** 2022-02-14

**Authors:** Maryam Ghorbani, Rasoul Yaali, Wolfgang I. Schöllhorn, Amir Letafatkar, Hassan Sadeghi

**Affiliations:** 1 Faculty of Physical Education and Sports Sciences, Kharazmi University of Tehran, Tehran, Iran; 2 Department for Training and Movement Science, Johannes Gutenberg-University Mainz, Mainz, Germany

**Keywords:** Model-oriented learning, Differential learning, Self-organized learning, Gait, Kinematics, Children

## Abstract

**Background:**

Lack of the neuromuscular control during locomotion in the knee joint leads to an increased risk of anterior cruciate ligament (ACL) injury in children. Hence, we aimed to explore the effects of a repetitive, model-oriented, and self-organized approach on lower limb kinematics during gait in children.

**Methods:**

In randomized controlled trial, 36 children with 4 ± 0.79 years of age from the children gym were randomly (a lottery method) allocated into three groups, including (1) the model-oriented (n = 10), (2) Differential Learning (n = 11), and (3) control (n = 10) groups. Kinematic data of hip, knee, and ankle joints in the sagittal plane were recorded by a GoPro camera at the moments of heel-ground contact and toe-off the ground before and after a 6-week intervention (two sessions per week).

**Results:**

The results indicate a 35% post-intervention increase of ankle dorsiflexion (95% CI: − 5.63 _ − 0.96) in the moment of heel-ground contact in the model-oriented group; however, knee flexion (95% CI: − 1.05 _ 8.34) and hip flexion (95% CI: 3.01 _ 11.78) were respectively decreased by 20% and 20%. After the intervention, moreover, ankle plantar flexion (95% CI: − 9.18 _ − 2.81) and hip extension (95% CI: − 12.87 _ − 3.72) have respectively increased by 37% and 37%, while knee flexion (95% CI: 3.49 _ 11.30) showed a %16 decrease in the moment of toe off the ground. As for the Differential Learning group, ankle dorsiflexion (95% CI: − 5.19 _ − 1.52) increased by 33%, and knee (95% CI: 0.60 _ 5.76) and hip flexion (95% CI: 2.15 _ 7.85) respectively decreased by 17% and 17% at the moment of the heel-ground contact following the intervention. At toe lifting off the ground, the plantar flexion (95% CI: − 7.77 _ − 2.77) increased by 35%, knee flexion (95% CI: 2.17 _ 7.27) decreased to 14%, and hip extension (95% CI: − 9.98 _ − 4.20) increased by %35 following the intervention for the Differential Learning group subjects. Based on the results obtained from the one-way ANOVA, there was a significant difference between these groups and the control group in all kinematic gait variables (*p* ≤ 0.05). However, no statistically significant differences were found between the two experimental groups.

**Conclusions:**

The results implied that the model-oriented repetitive and the self-organized Differential Learning approach were both appropriate to alter the kinematic gait pattern in the 3–5-year-old children. Previous research has almost exclusively recommended a model-oriented approach to change kinematic patterns and preventing non-contact motor injuries. However, the present study showed that the Differential Learning approach can help children to achieve the same goal by continuously changing environments and stimulating challenges.

*Trial registration*: Current Controlled Trials using the IRCT website with ID number of, IRCT20130109012078N5 “Prospectively registered” at 14/5/2021.

## Background

Children’s orthopaedic injuries are most common. These injuries often result in physical limits, adding to the burden on the victim and their immediate environment. Still, most injuries in young children go undiagnosed [1]. Non-contact Anterior Cruciate Ligament (ACL) rupture is thought to be caused by neuromuscular control breakdowns during dynamic motions. Thus, ACL injuries are associated to neuromuscular and motor control kinematic impairments [1–3]. Between 2005 and 2015, the rate of ACL reconstruction in children aged 5 to 14 tripled, from 10 instances (3.1%) to 310 (96.6%) [4]. There were 175 males (54.7%) and 145 girls (45.3%) affected. Girls (52%) had ACL injuries compared to boys (35%) [5]. Growing numbers of ACL injuries in children and adolescents are putting strain on the health system and affecting their development [1–5]. Unknown neuromuscular control and movement pattern development [5]. Injured ACLs benefit from correcting lower limb motor pattern and kinematics [5]. Correcting lower limb motor pattern and kinematics help prevent degenerative joint problems after ACL injuries [3].

The medial and lateral areas of the heel showed greater peak pressure during knee extension in people aged 20.4 ± 3.3. Increased COP-displacements in the anterior–posterior and medio-lateral directions were linked to reduced ankle plantar flexion and hip adduction. Heel and ankle eversion are considered risk factors for lower limb injuries when walking, running, or sprinting [4]. Foot striking causes internal tibialis tension, according to Willems et al. (2006). This over-applied force in such a structure causes the overuse injury [5]. Like Song et al. 2013 they pointed out that increased ankle eversion during gaiting increases the injury risk in lower limbs [5]. Hence, acquiring a stepping pattern that reduces risk factors is a primary demand to prevent falling and sudden stumbling during gating. Persch et al. (2009) concluded that improving gait kinematics and increasing joint range of motion during gaiting reduced the rate of falls [6]. Also, by improving the kinematic pattern of gait (absence of asymmetric gait), the metabolic energy consumed by the skeletal muscles during movement is reduced; so the movement becomes more efficient and economical [7, 8].

As children have less opportunity to exercise in safe conditions, learning a less injury-prone stride pattern should be a priority. It is vital to improve fundamental motor skills by assessing kinematic features and applying suitable interventions in children aged 3–5 years, as this is considered a critical developmental phase [9].

For motor skill learning, various approaches have been suggested. These approaches include prototype-oriented forms that are guided and taught by observational patterns [10], verbal instructions [10], multiple repetitions, and feedback, as well as learner-oriented ways that employ discovery learning and self-organization to educate the individual. Similar therapies should have similar benefits, according to the cognitive approach to rehabilitation and therapy. In view of the cognitive turn, this method views learning as a mental movement pattern that becomes more solidified through repetition of the same perceptual trace pattern. [11, 12]. This perspective thinks variability unsuitable for skill acquisition. Hence, the more similar the exercised pattern is to the prototype, the better the results [13]. The subject tries to internalise or automatize the learning process by repeating the prescribed movement pattern, and the pedagogue tries to increase learning and attractiveness of the activity by providing feedback to the learners [10, 13]. This method is often used in research to modify movement patterns to lower kinematic injury risk [14]. For example, Noehren et al. (2011) used prescriptions and feedback to teach a proper gait pattern to prevent hip adduction in the stance face, which causes patellofemoral discomfort. Running reduced hip adduction and pelvic drop. In one leg squats, hip internal rotation and adduction were reduced to 23% and 18% [14].

Recent learning theories focus on nonlinear causality, where little causes can have large consequences, and vice versa. Based on system dynamics and biomechanics. [15, 16]. Differential Learning (DL) [15–18] presented for the first time a practical strategy to take advantage of naturally existing variability in movement repeats [19, 20]. Contextual Interference method [21] allows for discrete variations that do not allow for continuous and random fluctuations (= errors). [21]. The DL approach viewed greater fluctuations within the distinct color of noise as the key source for learning and a potential source for commencing a self-organization process amplification [14, 22]. The distinction between “movement instructions” and “movement tasks” was proposed by reform pedagogues [23]. Whereas “movement instructions” prescribe the to-be-learned movement, “movement tasks” provide a broad framework within which the learner must solve a problem autonomously. A person can learn from their environment without repetition or corrective feedback in the most extreme version of the DL technique [19, 22]. This method has a good effect on brain activation [24, 25]. Less comparisons to stated ideals and less criticism result in less dissatisfaction for learners. Both increase learning pleasure, shown in brain activation. A holistic and systemic approach, DL does not artificially isolate the individual from their environment. Biomechanically, gravitational and inertial forces are continually at work, supplying critical neuromuscular content. To keep the neuro-muscular system adaptable, one must face obstacles. Because no two movements are same, the DL method considers variability necessary and relevant in practise [26, 27]. Whether a high-performance athlete's movement [18, 28] or an everyday action arising from millions of repetitions [14, 29, 30], movements have biomechanical distinctiveness. The brain system becomes more versatile, resilient to disruptions, and adaptable to tasks that fit the individual's talents.

By preventing extra training pressures applied on the individuals, precludes injuries and directs them to sustain their efforts in achieving their goals [31]. In some investigations, the effect of a DL-derivative on learning fundamental movement skills (FMS) was reviewed [32]. In this research, skills involving catching and throwing an object were assessed using the test of gross motor development-2 (TGMD-2) before and after intervention in children. The results showed that the average catching and overhead throwing scores have increased by 28% following the intervention [32]. Also, Mohamadi Orangi et al. (2021) compared the effectiveness of different pedagogical approaches in football on reducing the risk of kinetic and kinematic ACL injury factors in young boys [33]. Their results indicated advantages of the more variable approaches compared to the somewhat repetitive and prescriptive one in terms ofkinetic and kinematic variables [33]. Thus, the findings of this study and Gokeler's research suggest that different methods of learning and motor control should be considered while performing anterior cruciate ligament injury rehabilitation after sports injuries [34–36]. So that physiotherapists, occupational therapists, and doctors can successfully finish the post-injury rehabilitation process and limit the risk of re-injury [35, 36].

Adults have been the subject of most injury research. Most child research compare the effectiveness of learning modalities on fundamental sport skills. Only Axeti et al. (2017) investigated the effect of play on the kinematics of preschoolers' walking patterns [9]. Their intervention had a positive effect on the kinematics of the walking pattern [9]. But no other learning methods were compared. Obesity risk reduction and improvement in gait are expected with improved walking behaviour in children [34]. This study compares the effects of old and newer learning methods on kinematic gait patterns in children aged 3 to 5. Both methods should improve children's gait kinematics since, according to DL-theory [14], children can learn new exercises even when they are repeated. These fluctuations are akin to those introduced by enhanced variability training.

## Methods

### Study design

Prior to data collection, this study was approved by the research ethics committee of the Kharazmi University (Approval ID, IR.KHU.REC.1399.031). The protocol was prospectively registered on the IRCT website (ID number: IRCT20130109012078N5, date of first registration 14/5/2021). All participants’ parents were informed of the study procedures, and they signed an informed consent form obtained from a participant’s parent prior to participating, in accordance with the Declaration of Helsinki. In this study, the researchers and participant’s parent was Not blinded to the groups’ randomization and interventions receiving by participants.

In this study, lower limb kinematics in the moments of heel-ground contact and toe-off the ground as gaiting were considered a dependent variable. Also, a six-week repetitive and variable training was reckoned as an independent variable. The participants were recruited from children’s gyms.

### Participants

The research subjects included children aged 3–5 years old (with no history of lower limb injuries and underlying disease such as diabetes, cardiac and circulatory anomalies, chronic lung disease, Down syndrome, cancer & etc.) [the repetition-oriented group (height: 107.8 cm ± 2.39 cm, weight: 17 kg ± 1.56 kg); the self-organized group (height: 108 cm ± 2.40 cm, weight: 16.45 kg ± 1.50 kg); the control group (height: 107.9 cm ± 2.47 cm, weight: 16.70 kg ± 1.76 kg]. After their parents filled out the consent form, the children’s names were written on paper that were randomly placed in a box as in a lottery method. Then, the names were randomly picked up from the box. The first, second, and third names were assigned to the repetitive, self-organized, and control groups in the next stage. The rest of the names was assigned in the same sequence [37]. To minimize the impact between the groups, the children in the training groups were separated as much as possible, and the training times were arranged so that they could not meet. Using G-Power, the sample size was estimated at the significance level of 0.05, with a 0.85 statistical power and effect size of 0.3 (medium effect size), and repeated measurements evaluated the statistical method of analysis of variance. Moreover, 33 people were assigned into three groups, according to similar research [38], but considering the possible drop out in the sample subjects, 36 people were finally considered for this study (12 participants in each groups).

### Biomechanical testing

The digital camera Gopro Black 7 (model: Hero; Company: Gopro; country: USA) was used to assess the gait kinematics. The camera's location with shoots 2.7 k video at up to 120 frames per second was limited on the subjects’ side (in the sagittal plane and perpendicular to the frontal plane) with a 2-m distance. The Helen-Hayes method set the markers (anterior superior iliac spine, femur greater trochanter, lateral knee condyle, lateral malleolus, calcaneus bone, and fifth metatarsal bone).

Then, the subject was asked to follow the determined path. Since we cannot control the movement of 3- to 5-year-olds, children were asked to walk as fast as possible. Indeed, the subjects should take a 6-m path with a desirable speed. Given that kinematics and kinetics of motion are affected by speed [39], the pre-test and post-test movement speed was calculated using Kinovea software. Although the speed was different in the pre-test (1.21 ± 0.27) and post-test (1.27 ± 0.29), the difference was not statistically significant (*p* ≥ 0.05). Therefore, in this study, velocity changes were not a factor affecting kinematics. Subsequently, kinematic variables, including joint angles of hip flexion, knee extension, ankle dorsiflexion in the moment of heel-ground contact, ankle plantar flexion, knee flexion, and hip extension in the moment of toe off the ground were calculated using the Kinovea software [40, 41]. Hisham and et al. (2017) in their study that analysis of gait using Kinovea software, indicated that the obtained data of HD video Cam-Kinovea for the same subject under five trials weren’t statically significant difference (variance < 5%). So the HD video Cam-Kinovea is reliable system for analysis of gait [42]. According to the study of Adnan et al. (2018), the most average variances between outcome results of HD video cam-Kinovea and outcome results of an established infrared motion capture system (Hawk-Cortex) in the analysis of kinematics movement was less than 10%. It was concluded that the integration of HD video cam-Kinovea could become a valid motion capture-analysis system [40]. In the study of Puig-Divi et al. (2019), the kinematic data obtained by Kinovea software were compared with the AutoCAD software as the “golden standard” [43]. The angles obtained from Kinovea software were like the results of AutoCAD software with 95% confidence. The digitization error was minimized by minimizing the recording space to 2 m × 3.5 m. With a 2.7 k resolution of the video camera, this corresponds to an error of 1.1 mm per pixel without filters. For the data calibration in the Kinovea software, two cones with a height of 0.30 m and a cube with an edge length of 0.30 m were used We zoomed the images in by 250% to calculate the kinematic information in Kinovea software [43].

### Interventions

The children assigned into the experimental groups received the gait interventions for six weeks (to produce a sustained change in the gait pattern, 6 to 8 weeks are recommended to participate in the training) [44].

### Training protocols

#### Schedule for the interventions of both groups

- Duration of Intervention period: 6 weeks
- Sessions per week: two sessions per week
- Duration of a single session: 30 min (5, 20, and 5 min were respectively specified for warm-up, main session body involving gait pedagogy, and cool-down) were determined to perform each session.

#### Model-oriented training

The supposed correct gait pattern was first presented to the child (verbally and with a visual pattern), and the child was then asked to walk on the determined path. As a further matter, the subjects were provided with feedback [45].

Duration of intervention period, duration of sessions, and preparation for sessions: Same as the model group.

### DL-training group

As with the variable training in the sense of DL, each session has been considered based on particular purposes, a surrounding was designed, and the children were let walk on such a landscape according to the training presented in Table 1 [9].

**Table 1:** Training session for the self-organized learning group

Week	Training	Pictures
First week (session 1 and 2)	The regular pattern of gait was designed on the ground, and the child was then asked to take the determined path (to get accustomed to the environment)	
Second week (session 3 and 4)	Walking between the cones and blocks (to vary the width of the steps while walking)	
Third week (session 5 and 6)	Walking on an inclined (sloping) surface (to vary the heel-toe pattern during walking)	
Fourth week (session 7 and 8)	Walking on arranged tatami mats in different directions and a spiral form (to vary maintaining the gravity center and height as walking, and avoiding touching the tatami mat's outer surface for prevention)	
Fifth week (session 9 and 10)	Walking over obstacles placed on the course (to vary the appropriate flexion of the ankle, knee, and hip joints during the swing phase)	
Sixth week (session 11 and 12)	Children walking among the blocks, over the inclined surface and the obstacles arranged in a row (to vary all variations together with all that they have acquired during these six weeks)	

Duration of intervention, duration of sessions, and preparation for sessions: Same as the model group.

### Control group

The control group only participated in pre-and post-tests.

### Statistics

All data has been described based on mean and standard deviation. Deviation from Normal distribution was proofed using the Shapiro–Wilk test. Since the results obtained were higher than alpha (*p* ≥ 0.05), the distribution was normal. Since the necessary assumptions were not established to use the analysis of variance test with repeated measures (and as the M Box test, performs the Box's M-test for homogeneity of variance matrices obtained from multivariate normal data according to one grouping variable, was significant for some research variables), homogeneity of the variance matrices was not well observed. Further, the significance of some variables observed in Levene test shows that the assumption of between-group variance equality was not met. Finally, the results obtained from the Mauchly test indicated that this test was significant for some variables. So, the assumption of the within-subject variance equality has not been fulfilled. Therefore, a two-way MANOVA test was performed in which time (pre-test and post-test) and groups factors were considered as independent variables and measured kinematic factors were considered as dependent variables. The results of the M box test (*p* ≥ 0.05) showed that the homogeneity of matrix of covariances was established. In all dependent variables, the results of Levene test were not significant (*p* ≥ 0.05), which showed that the homogeneity of variance of the residuals of the model was established. Pillar Trace test was used as a result. Given that in the results of the two-way MANOVA test, group and time factors and their interaction were significant (Table 3); hence, to determine the difference between pre-and post-tests (within-group difference), the paired t-test was used (Table 4). To compare the groups, the results of the Univariate tests table of General Linear Model were presented along with the results of the LSD post hoc tests (Table 4). The significance level was deemed 0.05 for all the calculations. The effect size (r) was estimated for all comparisons [46] and classified as small effect (r = 0.10), medium effect (r = 0.30), or large effect (r = 0.50) [47]. All the statistical calculations have been conducted using SPSS 24 software.

## Results

The general characteristics of the subjects were presented based on the separated groups in the Table 2. There was no significant difference among the subjects of the three groups in terms of age, weight, and height of the children.

**Table 2:** Subjects’ general characteristics

Variables	Model oriented group (n = 10)	Self-organized DL group (n = 11)	Control group (n = 10)	*P* value
Age (Y.)	4 ± 0.81	4 ± 0.77	4 ± 0.81	0.91
Weight (Kg.)	17 ± 1.56	16.45 ± 1.50	16.70 ± 1.76	0.81
Height (cm)	107.80 ± 2.39	108 ± 2.40	107.90 ± 2.47	0.78
Body Mass Index (kg/cm^2^)	14.62 ± 0.39	14.09 ± 0.29	14.36 ± 0.53	0.65

*P* ≥ 0.05 means there is no significant difference among the groups

**Fig. 1:**
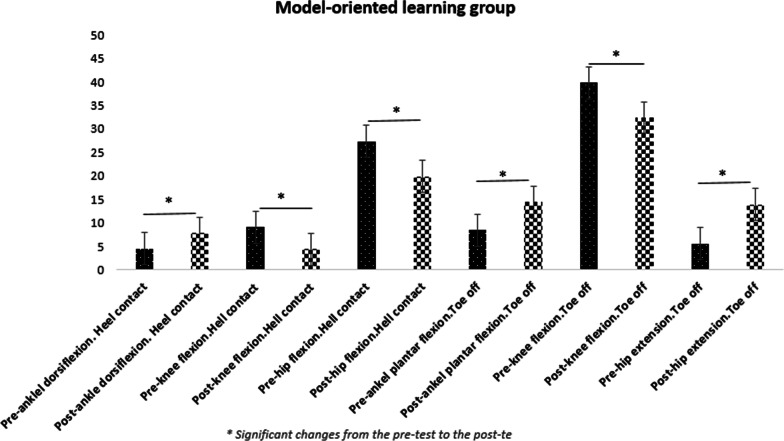
Changes in kinematic variables from pre-test to post-test in the model-oriented learning group

**Fig. 2:**
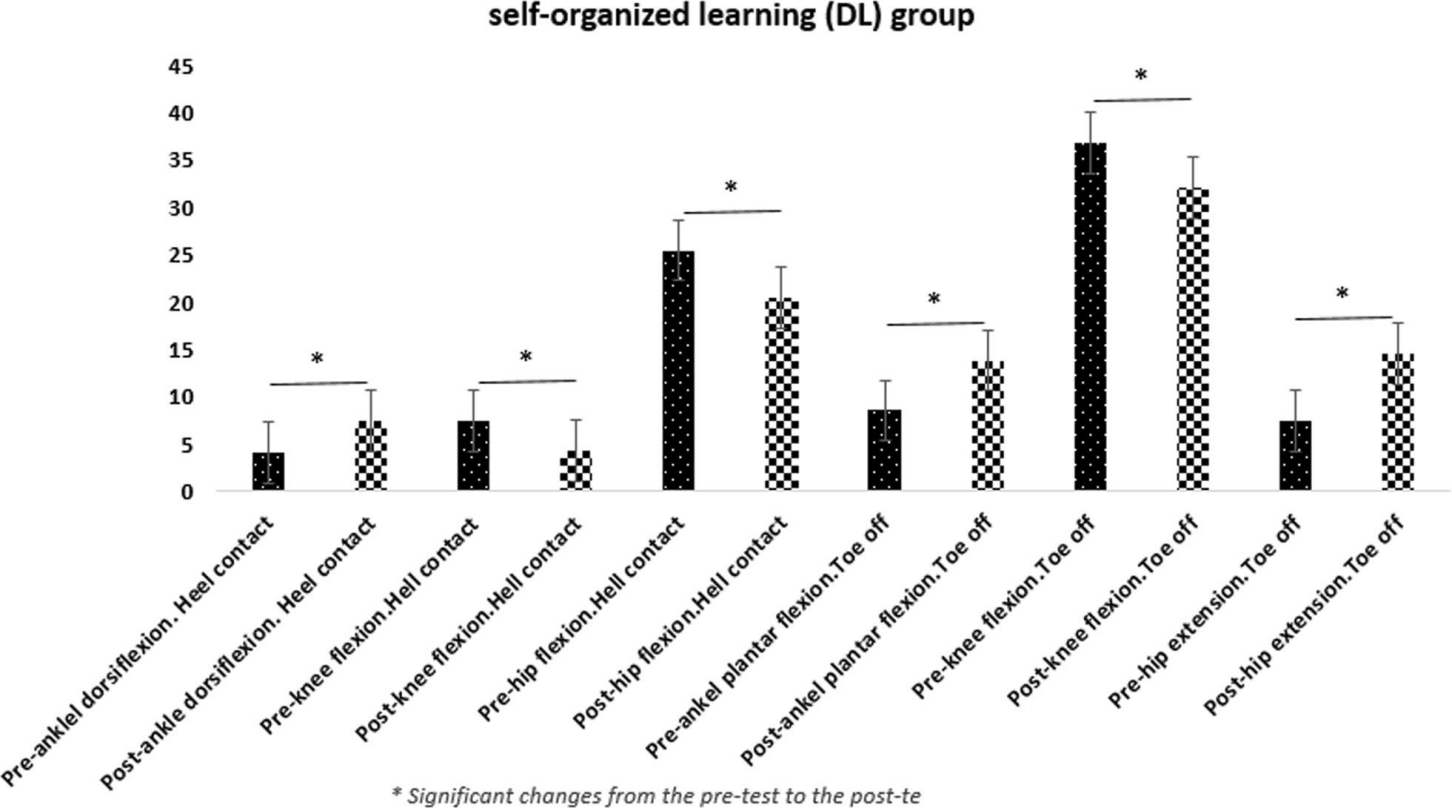
Changes in kinematic variables from pre-test to post-test in the DL group

**Fig. 3:**
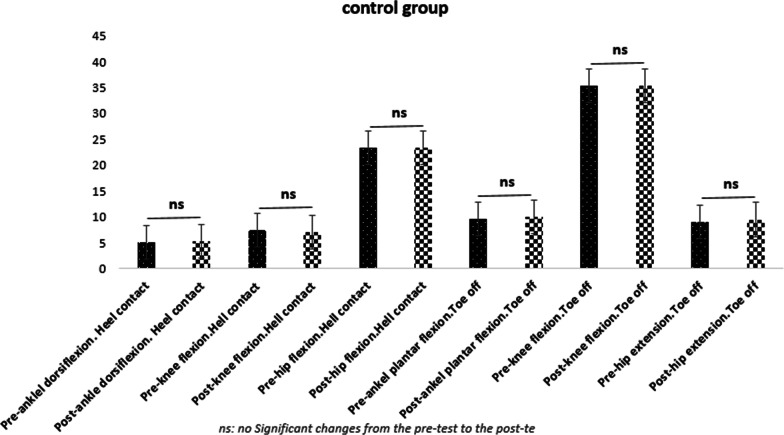
Changes in kinematic variables from pre-test to post-test in the control

**Fig. 4:**
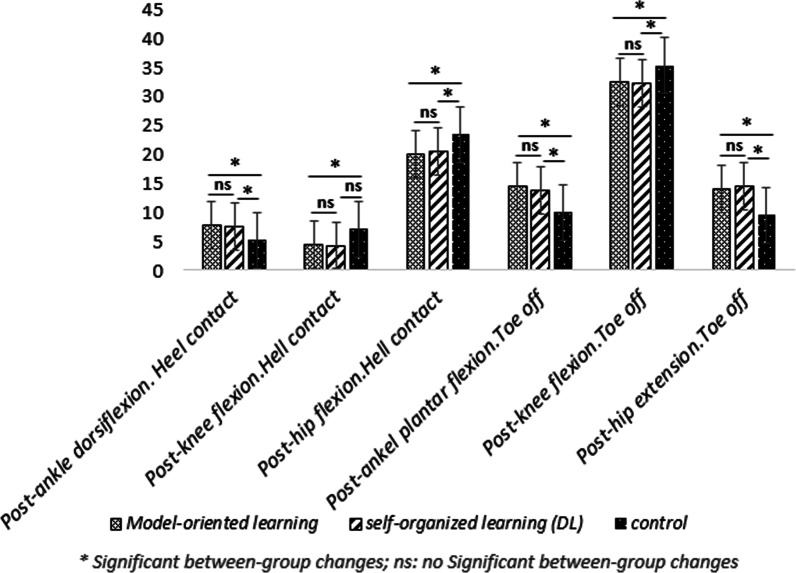
Comparison of changes in three groups from pre-test to post-test

Five participants discontinued the treatment prior to post testing due to personal reasons. Rates of compliance reported 88.6%. No adverse effects were seen during the study.The results of statistical analysis showed significant increases in the hip joint flexion angles [20% for repetitive (95% CI: 3.01 _ 11.78) and %17.76 for variable learning (95% CI: 2.15 _ 7.85)], knee flexion [20.62% for repetitive learning (95% CI: 1.05 _ 8.34) and 16.74% for DL (95% CI: 0.60 _ 5.76)], and ankle dorsiflexion [35.5% for repetitive learning (95% CI: − 5.63 _ − 0.96) and 33.85% for variable learning (95% CI: − 5.19 _ − 1.52)] were found out in the moment of heel-ground contact in both training groups in the post-test as compared to the pre-test. The joint angles of ankle plantar flexion [37.73% for repetitive learning (95% CI: − 9.18 _ − 2.81) and 35.56% for the variable learning (95% CI: − 7.77 _ − 2.77)], knee flexion [16.74% for repetitive learning (95% CI: 3.49 _ 11.30) and 14.83% for the variable learning (95% CI: 2.17 _ 7.27)], and hip extension [37.61% for the repetitive learning (95% CI: − 12.87 _ − 3.72) and 35.76% for the variable learning (95% CI: − 9.98 _ − 4.20)] indicated a significant difference in the moment of toe off the ground in the post-test compared to the pre-test (Table 3). The results obtained from the variance analysis test showed that there is a significant difference among the three groups (two experimental and one control) (Table 3). As a result, the LSD post-hoc test was conducted, and the related results were presented in the Table 4 (Figs. 1, 2, 3, 4).

**Table 3:** Results of the two-way MANOVA test

Effect	Value	f	Hypothesis *df*	Error *df*	*P* value	Partial Eta Squared
Intercept	0.99	1799.011	6	51	0.00	0.995
Group	0.454	4.71	12	104	0.04	0.23
Time	0.513	8.96	6	51	0.00	0.51
Group * time	0.344	2.79	12	104	0.03	0.31

**Table 4:** Within- and between-group changes

		Model-oriented learning	Self-organized learning (DL)	Control	Univariate tests of GLM	Posthoc LSD		
						Model oriented versus self-organized	Model-oriented versus control	Self-organized versus control
Ankle dorsiflexion at heel-ground contact (°)	Pre-test	4.5 ± 2.8	4.09 ± 3.7	5.00 ± 3.4	F = 8.90 d = 0.24 P = 0.00 MCID = 2.4°/1.6°	0.678	0.02¥	0.04¥
	Post-test	7.8 ± 2.1*	7.45 ± 2.1*	5.20 ± 3.5				
Knee flexion in the moment of heel-ground contact (°)	Pre-test	9.10 ± 7.8	7.45 ± 5.4	7.40 ± 7.8	F = 4.02 d = 0.12 P = 0.04 MCID = 8.48°/6.8°	0.64	0.04¥	0.09
	Post-test	4.40 ± 3.6*	4.27 ± 3.7*	7.10 ± 7.4				
Hip flexion in the moment of heel-ground contact (°)	Pre-test	27.30 ± 7.7	25.45 ± 5.1	23.40 ± 5.9	F = 8.31 d = 0.26 P = 0.006 MCID = 5.81°/2.8°	0.712	0.034¥	0.025¥
	Post-test	19.90 ± 4.8^*^	20.45 ± 4.5^*^	23.40 ± 5.4				
Ankle plantar flexion in the moment of toe off the ground (°)	Pre-test	8.40 ± 4.7	8.55 ± 3.0	9.60 ± 5.0	F = 14.26 d = 0.303 P = 0.00 MCID = 2.4°/1.6°	0.46	0.021¥	0.027¥
	Post-test	14.40 ± 2.9*	13.82 ± 2.8*	10.00 ± 5.2				
Knee flexion in the moment of toe off the ground (°)	Pre-test	39.80 ± 2.0	36.82 ± 4.6	35.30 ± 4.8	F = 13.81 d = 0.298 P = 0.00 MCID = 8.48°/6.8°	0.218	0.04¥	0.041¥
	Post-test	32.40 ± 53*	32.09 ± 3.7*	35.30 ± 4.4				
Hip extension in the moment of toe off the ground (°)	Pre-test	5.60 ± 5.2	7.45 ± 4.8	9.00 ± 7.0	F = 14.56 d = 0.306 P = 0.00 MCID = 5.81°/2.8°	0.462	0.037¥	0.040¥
	Post-test	13.90 ± 3.9*	14.55 ± 4.0*	9.50 ± 7.1				

^*^ Significant changes from the pre-test to the post-test in the significance level of 0.05

¥ Significant between-group changes in the significant level of 0.05

d = Partial Eta Square (Cohen’s D: the effect size value)

MCID = Minimal clinically important differences

GLM = General linear model

## Discussion

This study was aimed to investigate the effect of two learning approaches, including repetitive and variable learning, on the kinematic gait pattern in children ages 3–5 years old. The main result obtained in the study implied that both repetitive and variable approaches had positive effects on altering the kinematic pattern of lower limbs in walking.

The obtained results indicated that the repetitive approach could lead to a statistically significant effect on changes in ankle, knee, and hip joint angles at heel contacts the ground (Table 4). The present research results were consistent with those obtained in studies conducted by Persch [6] and Cao et al. [48]. This result may have happened because the target and to be tested gait pattern is emphasized in the repetitive approach, and the subjects were provided with feedback during walking to pay attention to the heel-ground contact. Therefore, this process led to increased ankle dorsiflexion and hip flexion, consequently decreasing knee flexion. In this regard, Noehren et al. [45] explored the effect of repetitive gait retraining based on real-time on hip kinematics in people with the patellofemoral pain syndrome. They used feedback during the retraining sessions to avoid hip adduction in the stance phase. Their results demonstrated that the hip adduction underwent a significant decrease in those with the patellofemoral pain syndrome [45]. So, providing subjects with feedback as performing related activities and emphasizing the correct pattern causes the movement kinematics to be improved. The movement also changed in two ankle and hip joints.

Because of a decrease in knee flexion, the repetitive approach will probably reduce the injury risk when walking. Moreover, the present results indicated that this approach significantly affects the ankle, knee, and hip joint angles at the toe-off the ground (Table 4). These results are also consistent with those obtained by Persch [6] and Cao et al. [48]. The plantar flexion of the ankle joint and the hip extension increase, and knee flexion decreases when toe-off the ground because the correct gait pattern is emphasized in the repetitive approach. The subjects are presented with feedback gaiting. It is emphasized to pay attention to the last point where the foot (i.e., toe) is off the ground and looks forward so that the body/trunk tends forward. As Noehren et al. [45] emphasized that the motor (movement) kinematics can be improved by providing individuals with feedback when they are moving, the movement pattern could also be modified, and the movement kinematics of gait could be enhanced by providing appropriate feedback in the a.

On the other side, the present study results showed that the variable approach has significantly affected ankle, knee, and hip joint angles (Table 4). The findings of the current study are consistent with those of Axeti et al. (2017), who found that the intervention significantly could improve the kinematics of the gait pattern. A possible explanation for this might be that manipulating surrounding conditions is one possibility of the principles of the Differential Learning theory on which the variable training was based to increase the amplitude of the joint kinematics [9]. Accordingly, the stimuli on the neurophysiological system were manipulated in this intervention to allow self-organization in the children’s gait patterns. It seems possible that these results are due to, the designed path, the obstacles, and the inclined surface were used to increase the variability of the heel-toe pattern so that the children were implicitly “forced” to increase the ankle’s plantar and dorsiflexion as well as the hip flexion [36]. In addition, they experienced a decrease in knee flexion at the moment of the heel-ground contact during downhill walking. This result may be explained by the fact that all the variables that characterize the supposedly correct pattern were performed without explicitly telling the children by designing corresponding exercise parkour [49]. Similar effects are intended by walking at different speeds or walking in curves, forward or backward. Some authors have speculated that all variations in walking led to changes in the kinematics of the gait and provided different stimuli to the muscular and proprioceptive system that fosters a reorganization of the whole movement organization in a more effective way [3, 9]. Therefore, it seems that the variable approach, which is partially based on continuously changing boundary conditions, may also be effective. However, it reduces the injury risk by inducing a more appropriate pattern and being prepared for eventual disturbances and the effect on the learning dynamics because of the increased experiences and more versatile activation of the protecting muscles. According to the present study, the results imply that the DL, in a broader sense, can have a significant effect on changes in ankle, knee, and hip joint angles when the toe takes off the ground (Table 4). These results are in agreement with Axeti et al. (2017) finding [9]. These factors may explain the boundary conditions of the surroundings were varied to induce self-organization and thereby finding an individually proper kinematic pattern so that an inclined surface, slalom walking, crossing obstacles, etc., was used to change, e.g., the heel-toe pattern. Consequently, they were implicitly forced to increase the amplitude in the ankle, knee, or hip joints [50]. By offering a multifaceted area, the appropriate pattern could be teased out by the children. In the context of self-organization, it is essential to note that the interventions were randomly applied in a diffuse and random increase of joint fluctuations and could have also been in many other sequences. Instead of teaching the children something or giving a new thing into the learning system, it is more like removing blockages within themselves to unleash skills they already have [18]. In a further step, the children could have been asked to create a form or path of locomotion by themselves too.

The post hoc variance analysis test results indicated no statistically significant difference between the two approaches regarding kinematic gait factors (Table 4). Although the repetitive approach has been emphasized in the literature exclusively to alter the kinematic movement pattern to prevent injuries [45], the present research results demonstrated that the appropriate pattern could also be acquired by most variable training according to DL theory. Although the logic also associates preventive aspects with the changed gait pattern, further research is needed to prove this. Other tests like dynamic balance tests for determining the reaction times could be one possibility [51].

Furthermore, the post hoc tests indicate a statistically significant difference between the repetitive and control group in all variables of the lower limb kinematic at the moments of the heel-ground contact and toe takes off the ground following the training intervention (Table 4). Whereas a strong trend in differences between the variable and the control group was seen in terms of the kinematic knee flexion in the moment of the heal-ground contact (Table 4), this kinematic factor may be likely improved considering increased ankle dorsiflexion and hip flexion within training sessions, particularly by increasing the slope for one session or providing more tasks with varying stride length. Therefore, these results are only partially consistent with the results found by Ghorbani Marzoni et al. [52] that funded that nonlinear pedagogy effective on the children performance. A significant drawback of all the designs that are based on exactly and explicitly training the target pattern with all the tested criteria, which is only plausible if the chosen movement pattern leads to what it promises, fewer injuries [45]. According to the most recent understanding of motor control, this can be doubted and proven by other transfer tests, e.g., walking speed in uneven terrain. However, manipulation was merely considered in the research carried out by Ghorbani Marzoni et al. [52] to compare the repetitive and variable approaches’ effectiveness. Hence, the present research implies that both approaches seem to have the same effectiveness in gait training in young children, inducing the appropriate movement and preventing non-contact injuries.

Nonetheless, some aspects need to be considered that could lead to an expansion of our understanding of the subject area. Because the sustainability of the interventions was not objective of research this should be investigated in future. A further aspect of interest would be the replicability of the observed phenomena with more sessions of intervention. Also differences related to the sex and socio-cultural background of the children would be of interest.

## Conclusions

The results indicated that both the repetitive and variable approaches can be appropriate for changing the kinematic gait pattern in children aged 3–5. However, the present research results provide evidence that the variable approach based on Differential Learning theory can provide the child with the appropriate pattern by offering a stimulating surrounding as well. The variable training also encourages children to move more and explore their body's reactions in communication with the discovery landscape, thereby enhancing their activity level and rate.

## Data Availability

The datasets used and/or analysed during the current study are available from the corresponding author on reasonable request.

## Abbreviation

95% CI: 95% Confidence interval of the difference (lower–upper)

## References

[1] Holden S, Boreham C, Delahunt E (2016). Sex differences in landing biomechanics and postural stability during adolescence: a systematic review with meta-analyses. Sports Med.

[2] Moksnes H, Grindem H (2016). Prevention and rehabilitation of paediatric anterior cruciate ligament injuries. Knee Surg Sports Traumatol Arthrosc.

[3] Lang PJ, Sugimoto D, Micheli LJ (2017). Prevention, treatment, and rehabilitation of anterior cruciate ligament injuries in children. Open Access J Sports Med.

[4] Song Q, et al. Biomechanics and injury risk factors during race walking. In ISBS-conference proceedings archive. 2013.

[5] Willems TM (2006). A prospective study of gait related risk factors for exercise-related lower leg pain. Gait Posture.

[6] Persch LN (2009). Strength training improves fall-related gait kinematics in the elderly: a randomized controlled trial. Clin Biomech.

[7] Peyré-Tartaruga LA (2021). Mechanical work as a (key) determinant of energy cost in human locomotion: recent findings and future directions. Exp Physiol.

[8] Peyré-Tartaruga LA, Coertjens M (2018). Locomotion as a powerful model to study integrative physiology: efficiency, economy, and power relationship. Front Physiol.

[9] Axeti G et al, Assessment of kinematic characteristics of preschoolers’ gait during the implementation of an intervention training program. 2017.

[10] Schmidt RA et al. Motor control and learning: A behavioral emphasis. Human Kinetics. 2018.

[11] Olaogun MO (1986). Adams' closed-loop concept of learning and motor performance: its application in behavioural kinesiology and patients' education in rehabilitation. Int J Rehabil Res.

[12] Adams JA (1987). Historical review and appraisal of research on the learning, retention, and transfer of human motor skills. Psychol Bull.

[13] Magill R, Anderson D (2013). Motor learning and control: Concepts and applications.

[14] Schöllhorn WI (2002). Identification of individual walking patterns using time discrete and time continuous data sets. Gait Posture.

[15] Schöllhorn W. Practical consequences of biomechanically determined individuality and fluctuations on motor learning. In: International society of biomechanics XVIIth congress. Calgary. 1999.

[16] Schöllhorn W (2000). Practical consequences of systems dynamic approach to technique and strength training. Acta Academiae Olympique Estonia.

[17] Schöllhorn WI. Konsequenzen der motorischen Entwicklung von Kleinkindern für das sportliche Training. In Österreichische Trainertagung Bad Aussee. 1999.

[18] Schöllhorn WI, Bauer HU. Identifying individual movement styles in high performance sports by means of self-organizing Kohonen maps. In: ISBS-conference proceedings archive. 1998.

[19] Schöllhorn WI (2009). Time scales of adaptive behavior and motor learning in the presence of stochastic perturbations. Hum Mov Sci.

[20] Schöner G, Haken H, Kelso JAS (1986). A stochastic theory of phase transitions in human hand movement. Biol Cybern.

[21] Shea JB, Zimny ST, Magill RA (1983). Context effects in memory and learning movement information. Memory and control of action.

[22] Dewey J (1916). Nationalizing education. Journal of Education.

[23] Mosston M, Ashworth S (1986). Teaching physical education.

[24] Henz D (2018). Post-task effects on EEG brain activity differ for various differential learning and contextual interference protocols. Front Hum Neurosci.

[25] Henz D, Schöllhorn WI (2017). EEG brain activity in dynamic health qigong training: same effects for mental practice and physical training?. Front Psychol.

[26] Bernstein N. The co-ordination and regulation of movements. The Co-ordination and Regulation of Movements, 1966.

[27] Hatze H (1986). Motion variability—its definition, quantification, and origin. J Mot Behav.

[28] Bauer HU, Schöllhorn W (1997). Self-organizing maps for the analysis of complex movement patterns. Neural Process Lett.

[29] Horst F (2017). Intra-individual gait patterns across different time-scales as revealed by means of a supervised learning model using kernel-based discriminant regression. PLOS ONE.

[30] Horst F (2019). Explaining the unique nature of individual gait patterns with deep learning. Sci Rep.

[31] Grigorenko EL (2020). Understanding, educating, and supporting children with specific learning disabilities: 50 years of science and practice. Am Psychol.

[32] Fahmi S, Jia Yi C. Impact of nonlinear pedagogy to teaching Fundamental Movement Skills (FMS). 2017.

[33] Mohammadi Orangi B (2021). Motor learning methods that induce high practice variability reduce kinematic and kinetic risk factors of non-contact ACL injury. Human Movement Science.

[34] Monte A. Insight into the biomechanics and bioenergetics of human walking: obese vs. healthy children. Exp Phys 2020. 10.1113/EP088772 32441828

[35] Gokeler A (2019). Principles of motor learning to support neuroplasticity after ACL injury: implications for optimizing performance and reducing risk of second ACL injury. Sports Med.

[36] Gokeler A (2019). Correction to: principles of motor learning to support neuroplasticity after ACL injury: implications for optimizing performance and reducing risk of second ACL injury. Sports Med.

[37] Gravetter FJ, Forzano L-AB, Research methods for the behavioral sciences.Cengage Learning. 2018.

[38] An H-J (2014). The effect of various dual task training methods with gait on the balance and gait of patients with chronic stroke. J Phys Ther Sci.

[39] Moissenet F, Leboeuf F, Armand S (2019). Lower limb sagittal gait kinematics can be predicted based on walking speed, gender, age and BMI. Sci Rep.

[40] Nor-Adnan NM (2018). Biomechanical analysis using Kinovea for sports application. IOP Conference Series: Materials Science and Engineering.

[41] Lund H (2008). A randomized controlled trial of aquatic and land-based exercise in patients with knee osteoarthritis. J Rehabil Med.

[42] Hisham NAH et al. Measuring ankle angle and analysis of walking gait using kinovea. 2017.

[43] Puig-Diví A (2019). Validity and reliability of the Kinovea program in obtaining angles and distances using coordinates in 4 perspectives. PLOS ONE.

[44] Shull PB (2013). Six-week gait retraining program reduces knee adduction moment, reduces pain, and improves function for individuals with medial compartment knee osteoarthritis. J Orthop Res.

[45] Noehren B, Scholz J, Davis I (2011). The effect of real-time gait retraining on hip kinematics, pain and function in subjects with patellofemoral pain syndrome. Br J Sports Med.

[46] Field A. Discovering statistics using IBM SPSS statistics. Sage. 2013.

[47] Cohen J. Statistical power analysis for the behavioral sciences. Academic Press. 2013.

[48] Cao Z-B (2007). The effect of a 12-week combined exercise intervention program on physical performance and gait kinematics in community-dwelling elderly women. J Physiol Anthropol.

[49] Wolfgang S, Fabian H (2019). Effects of complex movements on the brain as a result of increased decision-making. Journal of Complexity in Health Sciences.

[50] Shumway-Cook A, Woollacott MH. Motor control: translating research into clinical practice. Lippincott Williams & Wilkins. 2007.

[51] Michelbrink M, Schöllhorn WI (2005). 22.23 Differencial learning and random walk analysis inhuman balance. Gait & Posture.

[52] Ghorbani Marzoni M et al. The comparison of effectiveness Linear and Nonlinear Pedagogy on manipulation Motor Skills performance of children. Motor Behavior, 2019.

